# Strengthening health workforce capacity through work-based training

**DOI:** 10.1186/1472-698X-13-8

**Published:** 2013-01-24

**Authors:** Joseph KB Matovu, Rhoda K Wanyenze, Susan Mawemuko, Olico Okui, William Bazeyo, David Serwadda

**Affiliations:** 1 MakSPH-CDC Fellowship Program, School of Public Health, Makerere University College of Health Sciences, P.O. Box 7072, Kampala, Uganda; 2 Department of Disease Control and Environmental Health, School of Public Health, Makerere University College of Health Sciences, Kampala, Uganda; 3 Monitoring and Evaluation Technical Assistance (META) Project, School of Public Health, Makerere University College of Health Sciences, Kampala, Uganda

**Keywords:** Work-based, Health workforce development, Capacity building, Training, Uganda

## Abstract

**Background:**

Although much attention has been given to increasing the number of health workers, less focus has been directed at developing models of training that address real-life workplace needs. Makerere University School of Public Health (MakSPH) with funding support from the Centers for Disease Control and Prevention (CDC) developed an eight-month modular, in-service work-based training program aimed at strengthening the capacity for monitoring and evaluation (M&E) and continuous quality improvement (CQI) in health service delivery.

**Methods:**

This capacity building program, initiated in 2008, is offered to in-service health professionals working in Uganda. The purpose of the training is to strengthen the capacity to provide quality health services through hands-on training that allows for skills building with minimum work disruptions while encouraging greater involvement of other institutional staff to enhance continuity and sustainability. The hands-on training uses practical gaps and challenges at the workplace through a highly participatory process. Trainees work with other staff to design and implement ‘projects’ meant to address work-related priority problems, working closely with mentors. Trainees’ knowledge and skills are enhanced through short courses offered at specific intervals throughout the course.

**Results:**

Overall, 143 trainees were admitted between 2008 and 2011. Of these, 120 (84%) from 66 institutions completed the training successfully. Of the trainees, 37% were Social Scientists, 34% were Medical/Nursing/Clinical Officers, 5.8% were Statisticians, while 23% belonged to other professions. Majority of the trainees (80%) were employed by Non-Government Organizations while 20% worked with the public health sector. Trainees implemented 66 projects which addressed issues such as improving access to health care services; reducing waiting time for patients; strengthening M&E systems; and improving data collection and reporting. The projects implemented aimed to improve trainees’ skills and competencies in M&E and CQI and the design of the projects was such that they could share these skills with other staff, with minimal interruptions of their work.

**Conclusions:**

The modular, work-based training model strengthens the capacity of the health workforce through hands-on, real-life experiences in the work-setting and improves institutional capacity, thereby providing a practical example of health systems strengthening through health workforce capacity building.

## Background

Strengthening health systems is critical for achieving the global aspirations reflected in the Millennium Development Goals and other efforts to improve health outcomes [1,2]. Key among the efforts to strengthening health systems is the development of a committed, well-prepared, skilled, and knowledgeable public health workforce [3]. This can be achieved through training health workers to increase their knowledge and skills to provide quality health services. However, conventional training programs have not paid much attention on the role of skills building in health systems strengthening, or where this has happened; training programs have tended to focus on the theoretical rather than practical aspects of taught courses [4]. In addition, the tendency to bring health workers to centralized locations for training often causes serious disruptions in service delivery at facilities serving the most vulnerable populations [5]. Moreover, failure to reinforce skills and knowledge transfer by addressing other performance factors (such as work environment, organizational support, clear expectations and feedback, and motivation to reinforce proper attitudes and habits) have continued to hamper the effective application of newly-acquired learning in the workplace [4-6]. In addition, most training programs involve training of 1–2 individuals from an institution yet effective implementation of what is taught requires a multidisciplinary team working together as part of the health system [7]. These challenges suggest a need to implement alternative training programs that allow for interspersing acquisition of knowledge and skills with application in the working environment.

With this background in mind, Makerere University School of Public Health (MakSPH) with support from the Centers for Disease Control and Prevention (CDC) initiated an 8-month training program aimed at strengthening the capacity of health workers through work-based training. The main purpose of the training was to offer a training model that allows for knowledge and skills acquisition with greater involvement of other institutional staff for continuity and sustainability while causing minimum disruption to work processes. This approach makes the training to be contextual within the work place, using locally identified needs or gaps for hands-on training [4]. As Boud [8] noted, the defining characteristic of work-based training is that working and learning are mutually reinforcing since they take place concurrently. Learning is influenced by the nature of work and, in turn, work is influenced by the nature of the learning process that occurs [8].

Our work-based training program was modeled along the same principles as the CDC’s Data for Decision Making (DDM) program [9], the Master of Hospital & Healthcare Administration program in Ethiopia [10] and the in-serve capacity building program for family health in Brazil and Chile [11]. In tandem with these programs, our training program emphasizes implementation of an in-service project – between training modules – that necessitates the application of acquired skills to address significant health problems in the trainees’ place of work and mentoring of participants by experienced mentors. Mentors, including institutional supervisors at the place of work, serve not only as role models, but provide professional advice, feedback, and general support during the implementation of selected projects. This model is also partly related to the FETP principle of ‘learning by doing’ through its emphasis on work-based training [4,12], and provides a clear example of how the FETP approach can be adapted to health systems strengthening in resource limited settings. However, unlike the FETP where trainees spend 60-70% of the training period in the field [12], the focus of our training program is building institutional capacity through training of individuals (who are full-time employees) at a particular institution, and addressing organizational challenges through hands-on training.

The purpose of this paper is to share our experiences in implementing a work-based, in-service training model aimed at developing and strengthening the capacity/competences of health workers to deliver quality health services in Uganda. The experiences shared in this paper are based on our interaction with the trainees during the course of the training, visits to the work-place where trainees are based, and analysis of routine program data (including basic demographic and background training information collected from the trainees at the time of registration, and documentation of Fellows’ progress and projects). A comprehensive evaluation of the impact of this program is in the pipeline and is thus not the focus of this paper, which mainly addresses the training processes.

## Methods

### Setting

MakSPH is one of the four Schools of Makerere University College of Health Sciences, a constituent College of Makerere University in Kampala, Uganda. The mission of the School is to improve the attainment of better health for the people of Uganda through public health training, research and community service while its vision is to be a center of excellence providing leadership in Public Health. The training program described in this paper was implemented by MakSPH-CDC Fellowship Program based at MakSPH.

### Program context

The main objective of the Fellowship program is to provide systematic public health training focused on increasing the number of professionals equipped with program leadership and management skills to implement health programs, strengthen and/or replicate successful health interventions, as well as enhance the sustainability of health programs in Uganda. Established in 2002, the program initially focused on the long-term Fellowship program; a 2-year, non-degree, full-time program that is offered to Ugandan nationals with a Master’s degree in health-related fields. The MakSPH-CDC Fellowship Program has been described elsewhere [13]. In the long-term Fellowship, individuals are placed at other organizations (not their original work place) for apprenticeship. However, there was growing interest in training individuals within their work place in order to link acquisition of knowledge and skills in leadership and management to addressing specific contextual institutional management-related problems. Furthermore, many organizations could not afford to have their staff away from work for such a long period. This led to the emergence of this modular, work-based training program. It was developed to offer an alternative training approach which allows trainees to identify priority problems within their work places and implement simple, but focused projects meant to address them.

### Work-based training program

Initiated in 2008, the work-based program is an apprenticeship training program in which trainees (“Fellows”) work closely with academic mentors (selected from MakSPH faculty and the training team) and institutional supervisors to accomplish specific tasks while continuing with their employment. Trainees are most often full-time employees who are given time away from their jobs to attend the face-to-face interactive portion of the program and return to their posts for the practicum component. To facilitate effective implementation, MakSPH-CDC Fellowship Program signs a Memorandum of Understanding with the head of each institution whose staffs have been selected to participate in the training. This acts as a form of commitment for both institutions to support the trainee with respect to attendance of face-to-face sessions, project implementation, and mentorship support as required.

The program is offered in two tracks: monitoring and evaluation (M&E) and continuous quality improvement (CQI). Twenty four trainees from 12 institutions (i.e. two trainees per institution) are admitted per course to insure continuity in the event that one of them drops out of the program and to enhance involvement of other institutional staff. Trainees are encouraged to work with other people as part of a team. The focus on M&E was informed by the realization that many institutions still face challenges in producing data of sufficient quality to permit the regular tracking of progress in improving and scaling-up health interventions [14]. On the other hand, the focus on CQI was based on the realization that while modern approaches to improving quality are increasingly used globally [1], their adoption remains sporadic in most developing countries, Uganda inclusive [15]. As Leatherman et al. [1] have pointed out, demonstrable improvements in quality of health care, coupled with sound monitoring and evaluation systems may in fact encourage greater investment in health systems strengthening by increasing donor, population and governmental confidence that resources are being used more effectively and efficiently.

### Training approach

**Figure 1:**
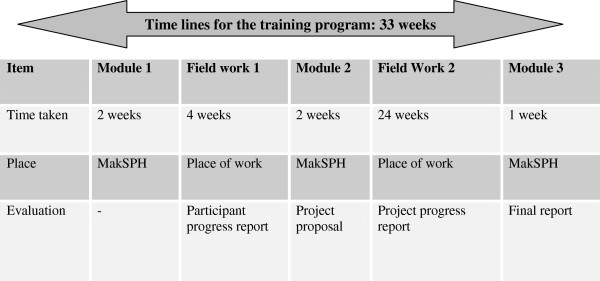
Schematic illustration of the training program.

The program is offered to mid- and senior-level managers, coordinators and supervisors who can influence systems and improve management processes and leadership practices within their institutions. Trainees participate in short face-to-face sessions (for a maximum of two weeks at any time) at MakSPH and return to their institutions between modules to try out what they have learnt in class, thereby minimizing boredom and work-place disruptions. The training is arranged in three modules that are spread throughout the 8-month duration of the training. The first and second modules run for a period of two weeks each while the third module runs for one week (Figure 1 and Table 1). The first module introduces trainees to the concepts in M&E or CQI. It prepares them to start thinking about a problem or gap within the organization that they intend to address as part of the training. This constitutes the trainees’ hands-on ‘project’. After the first module, trainees return to their place of work to concretely identify and refine their projects, working jointly with other institutional staff as part of a team. Working as part of a team in problem identification helps to increase ownership of the project and builds the capacity of other institutional staff to use similar processes in solving related problems in future. Trainees’ projects should address existing priority institutional work-related weaknesses or problems and should be implemented within the mandate of the institution. The projects are reviewed and cleared by the institution’s leadership and this increases prospects for project continuity and scale-up to other units. Trainees have up to one month to identify and refine their projects before they return to MakSPH to attend the second module.

**Table 1:** Modular presentation of the course schedules

**Modular schedules**	**M&E**	**CQI**	**Remarks**
Module I	· Introduction to M&E concepts	· Background to CQI	Trainees are introduced to the basic concepts pertaining to the track undertaken and to the basic tenets of problem identification & definition
	· Designing M&E systems	· Seven-step problem solving process	
	· Logical & results framework	· Flow analysis	
	· Program monitoring		
	· Evaluation design		
	· Monitoring and evaluation plan		
	· Project proposal writing		
Module II	· Data management	· Managing the project	Trainees present concept papers on problem areas identified and are supported to develop them into full proposals. Trainees have up to 24 weeks between Module II and Module III to implement their proposed project.
	· Data analysis		
Module III	· Report writing	· Strategic communication	Trainees return for the last module and present project progress reports on projects undertaken. Trainees also receive instruction on how to disseminate their final project reports
	· Communication	· Presentations	
	· Presentations		

During the second module, trainees from each institution present their proposed project to a team composed of Fellowship program staff and trainers at MakSPH who review the projects prior to full proposal development. Trainees continue to engage in face-to-face interactive sessions during the second module and finalize their project proposals before they return to their institutions.

Between the second and the third module, trainees have up to six months of project implementation time and receive up to $2500 (two thousand five hundred dollars) from MakSPH-CDC Fellowship Program to support project implementation. This money is channeled through the institution account. Depending on need, institutions supplement this budget to facilitate trainees to implement changes needed to achieve set targets. Only one project is supported per institution and trainees from the same institution work together on the same project as part of a team.

At the beginning of each training program, pairs of trainees are assigned academic mentors. An academic mentor is a member of staff of MakSPH or one of the facilitators whose role is to facilitate, guide, and support the trainee in problem identification & prioritization, proposal development, project implementation and report writing. At each institution, one staff, usually the head of the institution, or the head of the department to which the trainees belong, is designated as an institutional mentor. The institutional mentor facilitates the trainees’ learning by providing them with permission to attend the face-to-face interactive sessions, topping up the project grant if needed, and by supporting ownership and institutionalization of the project implemented. During the course of the training, trainees receive on-site and off-site supervisory support from the academic mentors and from Fellowship program staff. This support is maintained till completion of the training. Currently, no post-training follow-up support is provided to the trainees; an area that is being evaluated.

At the end of the project, trainees return to MakSPH for the third module. Trainees present progress reports on the projects implemented and receive guidance on how to communicate the project results including report writing and presentations. Trainees write and submit final project reports that describe the status of the project at the time, the improvements achieved, lessons learned, challenges experienced and those that still exist, as well as any plans for scaling up lessons learnt/best practices identified. In addition, all trainees participate in a dissemination workshop organized by the Fellowship Program to share their project reports with other stakeholders. The workshop is attended by institutional representatives, alumni trainees, facilitators, mentors and other stakeholders.

### Monitoring and evaluation of the program

As part of the training, trainees receive up to three on-site visits to check on the progress of project implementation and assess the level of project integration into other institution’s activities. During such visits, discussions are held with the management of the institution with regard to the support available to the trainees to complete the projects and any prospects for scale-up/continuity of project activities initiated by the trainees beyond the current unit/department where they are based. As part of the routine visits, mentors and program staff discuss issues pertaining to the challenges experienced by trainees during project implementation and how the trainees tried to resolve them. If any major challenges still exist at the time of the visit, these are brought to the attention of management. These visits continue until the end of the training period. At the final dissemination workshop, a trainees’ representative makes a presentation on the benefits that accrued from participating in the program, the challenges experienced during the training and through project implementation; how these challenges were minimized, and any prospects for project sustainability at institutional level. After the dissemination workshop, exit interviews are administered anonymously to obtain additional insights and trainees’ reflections on the program. Some of these insights have been reproduced in this paper.

## Results

**Figure 2:**
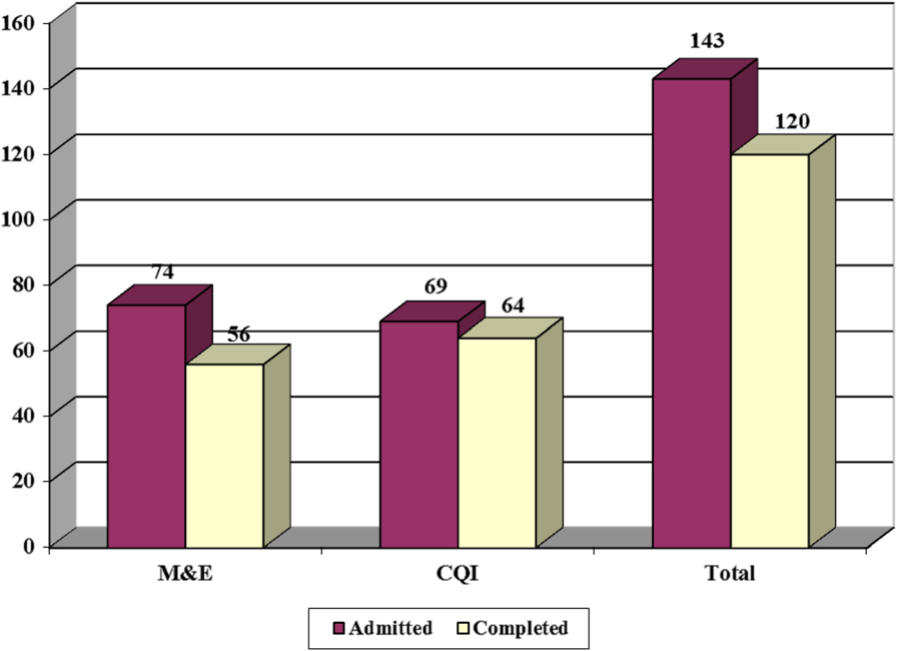
Number of trainees admitted and who completed the training: 2008–2011.

Overall, 143 trainees were admitted between 2008 and 2011. Of these, 120 (84%) trainees from 66 institutions completed the training successfully (Figure 2). Of the 120 trainees, 44 (37%) were Social Scientists; 41 (34%) were Medical/Nursing/Clinical Officers; 07 (5.8%) were Statisticians while 28 (23%) belonged to other professions. Majority of the trainees (80%) were employed by Non-Government Organizations (NGOs) while 20% worked with Government Ministries/Departments, district health services and hospitals. Of the 120, 56 (46.7%) were enrolled for M&E while 64 (53.3%) were enrolled for CQI. Among those enrolled for M&E, completion rates improved from 59% (13/22) in 2008 to 88.2% (15/17) in 2011. In those enrolled for CQI, completion rates improved from 91.7% (22/24) in 2008 to 100% (12/12) in 2010 but dropped slightly to 88.2% (15/17) in 2011. Overall, training completion was higher among those enrolled for CQI (64/69, 92.8%) than those enrolled for Monitoring and Evaluation (56/74, 75.7%). Majority of the M&E trainees who did not complete the training took jobs with other institutions mid-way the training given the high demand for skilled M&E personnel in Uganda.

### Project outputs/outcomes

Altogether, trainees implemented 66 projects (32 in M&E and 34 in CQI) between 2008 and 2011. These included projects aimed at strengthening M&E systems; improving data management and reporting; improving performance tracking systems; reducing the proportion of clients who missed scheduled clinic visits/services; improving tuberculosis (TB) diagnosis and treatment; and streamlining client appointment systems, among others (Table 2 and Table 3). Nearly half of the M&E projects (14 of 32) were on strengthening of M&E systems, reflecting a critical need for strengthening M&E systems in Uganda. Thirteen of the CQI projects were on interventions aimed at reducing the time patients take to access services as well as ensuring timely reporting (e.g. submission of reports from the district to the Ministry of Health) while the remaining 21 projects addressed other bottlenecks in health service delivery including decreasing the proportion of patients lost to follow-up in ART and TB programs; improving tracking of clients’ medical charts; improving TB treatment completion rates; and improving the procurement process for ART supplies, among others.

**Table 2:** Examples of projects implemented by trainees

**Project title**	**Project timeframe**	**Aim**	**Outputs/Outcomes**
Reducing patient waiting time for clients attending the ART clinic at a regional referral hospital	September 2008 – February 2009	To reduce the patient waiting time to receive ART services at the clinic from six hours to four hours so as to improve quality of health care services at the clinic.	· The average waiting time at the ART clinic reduced from 348 minutes to 220 minutes, a 36.8% reduction in overall clinic waiting time.
			· Specifically, there was also a 42.4% reduction in waiting time at the Clinician & Laboratory stations (waiting time reduced from 257 minutes at baseline to 148 minutes at the end of the project)
Consolidating the M&E System at a local NGO	October 2008 – March 2009	To contribute to the continuous improvement of the M&E function through consolidating the M&E system	· Defined the purpose and scope of the M&E system
			· Developed the M&E Matrix
			· Reviewed the logical framework
			· Reviewed report formats
			· Documented the critical reflection schedule
			· Designed a schematic presentation of the M&E system
			· Defined indicators & documented indicator reference sheets
Enhancement of the performance tracking system at a government department	September 2009 – February 2010	To improve the performance tracking system for effective tracking of programme performance	· Developed an M&E coordination mechanism
			· Developed the M&E System
			· Harmonized and agreed on key performance indicators
			· Developed M&E Matrix
			· Developed the Results Chain
Reducing the turn-around time for voluntary counseling and testing (VCT) clients at a University-based health facility	October 2009 – March 2010	To reduce turn round time for VCT client by 50%, thus improving access to and use of VCT services by staff and students	· Turn-around time for VCT reduced by 48%, from 122 to 63 minutes
			· There was a 10-fold increase in number of testers
			· VCT record handling was streamlined
			· Team spirit was enhanced
			· Staff are more kin on documentation of activities than before
Improving quality of HIV/AIDS and related data at a district local government	September 2010– February 2011	Improve completeness of in-patient reporting from the district to the Ministry of Health from 53% to 85% & out-patient reporting from 84% to 100%	· In-patient reporting completeness improved from 53% to 82.8%
		Improve timeliness of reporting from the district to Ministry of Health from 75% to 100%	· Outpatient reporting completeness improved from 84% to 96.5%
			· Timeliness of reporting improved from 75% to 80%
			· Improved district ranking in national district league table from 61^st^ to 45^th^ during the project period.
Enhancing data management by improving the client identification system at a local NGO	October 2010 – March 2011	Improve data management through improving the client identification system	· The proportion of client charts with one consistent identification number increased from 11% to 100% during the project period
			· The proportion of clinic charts with two numbers decreased from 61% to 0%

**Table 3:** Summary of projects implemented by medium-term fellows, 2008 – 2011

**Track/Category**	**Number of projects**	Nature of projects implemented (number of projects indicated in brackets)*	**Examples of project outputs**
**Monitoring and Evaluation Track**			
Establishing/strengthening the M&E system	15	· Strengthening M&E systems (13)	**Project title**: Consolidating the M&E System at a local non-government organization
		· Establishing M&E system (1)	**Aim**: To contribute to the continuous improvement of the M&E function through consolidating the M&E system
		· Developing M&E plan (1)	**Project time-frame**: October 2008 – March 2009
			**Key Outputs**:
			· Defined the purpose and scope of the M&E system
			· Developed the M&E Matrix
			· Reviewed the logical framework
			· Reviewed report formats
			· Documented the critical reflection schedule
			· Designed a schematic presentation of the M&E system
			· Defined indicators & documented indicator reference sheets
Improving data management and reporting	13	· Improving data management and quality of data collected (7)	**Project title**: Improving quality of HIV/AIDS and related data at a District Local Government
		· Improving the reporting system (4)	**Aim**:
		· Strengthening health data & information management systems (2)	· Improve completeness of in-patient reporting from the district to the Ministry of Health from 53% to 85% & out-patient reporting from 84% to 100%
			· Improve timeliness of reporting from the district to Ministry of Health from 75% to 100%
			**Project time-frame**: September 2010- Feb 2011
			**Key Outputs**:
			· In-patient reporting completeness improved from 53% to 82.8%
			· Outpatient reporting completeness improved from 84% to 96.5%
			· Timeliness of reporting improved from 75% to 80%
			· Improved district ranking in national district league table from 61^st ^to 45^th ^during the project period.
Other M&E-related areas	04	· Enhancing the performance tracking system of reproductive and HIV/AIDS care programs (3)	**Project title**: Enhancement of the performance tracking system of the Reproductive Health Component of a donor-funded program at a government department
		· Strengthening the community component of an organization’s M&E system (1)	**Aim**: To improve the performance tracking system for effective tracking of programme performance
			**Project time-frame**: September 2009 – February 2010
			**Key Outputs**:
			· Developed an M&E coordination mechanism
			· Developed the M&E system
			· Harmonized and agreed on key performance indicators
			· Developed M&E Matrix
			· Developed the Results Chain
**Continuous quality improvement**			
Reducing proportion of clients who miss scheduled clinic visits or services	10	· Reducing waiting/turn-around time for clients to receive HIV prevention, care and treatment services (5)	**Project title**: Reducing patient waiting time for clients attending the ART clinic at a regional referral hospital
		· Reducing the proportion of clients who miss their scheduled clinic visits (2)	**Aim**: To reduce the patient waiting time to receive ART services at the clinic from six hours to four hours so as to improve quality of health care services at the clinic.
		· Improving availability of staff on duty to provide ART services (1)	**Project time-frame**: September 2008 – February 2009
		· Decreasing the proportion of patients lost to follow-up (1)	**Key Outputs**
		· Improving tracking of clients’ medical charts (1)	· The average waiting time at the ART clinic reduced from 348 minutes to 220 minutes, a 36.8% reduction in overall clinic waiting time.
			· Specifically, there was also a 42.4% reduction in waiting time at the Clinician & Laboratory stations (waiting time reduced from 257 minutes at baseline to 148 minutes at the end of the project)
Improving data collection, management and reporting	06	· Improving HIV care/ART/OVC data collection & reporting (2)	**Project title**: Enhancing Data Management by improving the client identification system at a local NGO
		· Improving the data management system (2)	**Aim**: Improve data management through improving the client identification system
		· Improving the quality of program reports (2)	**Project time-frame**: October 2010 – March 2011
			**Key Outputs**:
			· The proportion of client charts with one consistent identification number increased from 11% to 100% during the project period
			· The proportion of clinic charts with two numbers decreased from 61% to 0%
Improving TB diagnosis and treatment	04	· Improving Tuberculosis (TB) treatment success rate (1)	**Project title**: Reducing the proportion of TB suspects bringing less than 2 sputum samples at a local NGO
		· Improving TB diagnosis among persons living with HIV (1)	**Aim**: To increase the proportion of TB suspects bringing 2 sputum samples from 40% in November 2009 to 90% by April 2010
		· Reducing the number of TB suspects bringing less than 2 sputum samples (1)	**Project time-frame**: November 2009 – April 2010
		· Reducing patient waiting time to initiate TB treatment at a Regional Referral Hospital (1)	**Key outputs:**
			· Proportion of TB suspects bringing 2 sputum samples increased from 40% to 83% in April 2010, and reached 97% by July 2010
			· Reduction in waiting time from 6 to 2 hours
			· TB infection control measures were strengthened
			· TB suspects’ and patients’ follow-up improved during the project period
Improving capacity for program staff and community volunteers to deliver HIV services	03	· Improving the capacity of community care givers to provide quality Positive Health, Dignity and Prevention services among HIV-positives (1)	**Project title**: Enhancing the capacity of a local non-government organization’s staff to develop and deliver effective health messages for adolescents
		· Enhancing capacity of a local non-government organization’s staff to develop and deliver effective health messages to adolescents (1)	**Aim**: Increase the proportion of staff with knowledge and skills in message development from 45% to 80%.
		· Increasing the number of planned faithful house trainings for married persons implemented at a donor-funded Project (1)	**Project time-frame**: October 2011 – March 2012
			**Key outputs**:
			· The proportion of staff with skills in effective message development and delivery increased from 45% at baseline to 75% at the end of the project
			· Staff employed a multi-channel approach in the delivery of messages
Streamlining appointment system	02	· Streamlining patients appointment system at a local HIV/AIDS Initiative (1)	**Project title**: Improving the proportion of clients honoring their appointments at an HIV care and treatment facility
		· Improving the proportion of clients honoring their appointments (1)	**Aim**: To improve the proportion of clients honoring their appointments from 48.2% to at least 80% in six months
			**Project time-frame**: March – September 2011
			**Key outputs**:
			· Proportion of clients keeping appointments from 48.2% to 81%
			· Developed standard operating procedures for clinicians emphasizing the importance of appointments’ keeping
			· Redesigned the clients’ prescription form to include ‘next appointment date’ and reason for the visit
			· Designed weekly appointment schedules and circulated them to all clinicians
			· Developed information, education & communication (IEC) messages and materials emphasizing the importance of keeping appointments
			· Designed a patient flow chart to guide patient flow
			· Conducted daily health education sessions where the issue of appointments’ keeping was emphasized
Improving proportion of individuals utilizing HIV prevention services	02	· Increasing male circumcision uptake among referred study participants (1)	**Project title**: Increasing the proportion of medical male circumcision uptake among study participants at a research site
		· Increasing the proportion of counseled clients tested for HIV at an outreach site (1)	**Aim**: Increase uptake of medical male circumcision among study participants from 20 to 34%
			**Project time-frame**: March – September 2011
			**Key outputs**:
			· Uptake of medical male circumcision increased from 20.5% at baseline to 32.4% at the end of the project
			· Two male circumcision notification strategies were used: phone contact and physical re-notification. Phone contact notification was found to be cheaper than physical re-notification
Improving psycho-social support for people living with HIV	02	· Strengthening psychosocial support through increasing enrollment and participation of children in Kids’ Clubs in Kigarama sub-County, Sheema District (1)	**Project title**: Increasing participation of psychosocial groups in PMTCT activities through couple-to-couple strategy at a local NGO
		· Increasing enrolment and participation of psychosocial groups in Prevention of Mother-to-Child HIV Transmission activities in Kabwhole-Itendero Town Council, Sheema District (1)	**Aim**: Increase the general enrolment into psychosocial groups from 47% to 70% by the end of the project
			**Project time-frame**: February – August 2012
			**Key outputs**:
			· General enrolment into psychosocial groups increased from 47% to 87% by August 2012
			· Proportion of male partners participating in PMTCT activities increased from 21% to 30%
			· Trained 16 model mentor couples to provide outreach PMTCT services in the community
			· Built capacity of psychosocial groups to support community members in coping with the effects of HIV/AIDS
			· Strengthened referral systems between the community and health facilities.
Improving access to Prevention of Mother-to-Child Transmission (PMTCT) of HIV services	02	· Improving access to comprehensive PMTCT services at a Regional Referral Hospital (1)	**Project title**: Improving linkage to PMTCT at a private-not-for-profit hospital
		· Improving linkage to PMTCT care at a private-not-for-profit hospital (1)	**Aim**: To improve linkage to PMTCT for HIV-positive pregnant mothers attending antenatal care services from 40% to 70%
			**Project time-frame**: August 2011 – January 2012
			**Key outputs**:
			· Proportion of HIV-positive pregnant mothers linked to PMTCT increased from 40 to 78.2%; 95% of these mothers got ARVs
			· Time for provision of PMTCT services at the antenatal care clinic declined from 7 to 4.5 hours
Other CQI-related areas	04	· Increasing the proportion of eligible clients initiated on antiretroviral therapy (ART) at a government health center in Jinja district (1)	**Project title**: Increasing the proportion of eligible clients initiated on ART at a government health center in Jinja district
		· Improving the procurement process for ART supplies at a local HIV/AIDS Initiative (1)	**Aim**: To increase the proportion of eligible clients initiated on ART from 26.7% in August 2008 to 75% by January 2009
		· Improving ART adherence assessment at an HIV clinic at Mulago Hospital, Kampala, Uganda (1)	**Project time-frame**: August 2008 – January 2009
		· Improving efficiency and quality of home visit services at a faith-based hospital in Kampala, Uganda (1)	**Key outputs**:
			· By April 2009, all eligible and pending clients attending the clinic were initiated on ART
			· ART logistics orders were made timely and no stock outs were reported during the project period
			· Two clinic days were established with each clinic designated to see 25-30 patients per day compared to 60 patients before the project: this increase the client-provider interaction time
			· Increased monthly attendance at the clinic from 162 in October 2008 to 253 by April 2009.

*Detailed presentations of all projects indicated in this table are available at the Fellowship Program website at: http://www.musphcdc.ac.ug.

At the final dissemination workshop, and through exit interviews, majority of the trainees expressed appreciation for the program and emphasized that their skills and competencies were enhanced as a result of participating in the training, as the following quotations suggest:

*“The quality of Orphans and Vulnerable Children (OVC) data collected during and after the project has tremendously improved; the errors are very minimal compared to the period before the project” ****CQI trainee***.

*“Knowledge and skills of community lay persons improved since they can now meet deadlines. Before 40% could report by the date of submission today 90% do submit their reports on time each month…” ****M&E trainee***.

*“We were able to increase the proportion of clients who received their initial CD4 screen test (base-line). Many of them were old clients – as old as one year in the palliative system” ****CQI trainee***.

*“The organization has a better understanding of M&E and appreciates why the organization has been unable to track results” ****M&E trainee***.

## Discussion

Our experiences suggest that hands-on, work-based training can be a useful model for strengthening health workforce capacity/competence for improved health service delivery. This approach can also directly address existing challenges in strengthening health systems at the work place. Our training program builds trainees’ capacity to critically analyze work and management processes as well as systems through the initial face-to-face interactive sessions. Thereafter they have an opportunity to actually identify, implement and evaluate projects meant to improve the quality of health services provided by their institutions, albeit using M&E and CQI as the foci. The trainees’ competences are further augmented through the off-site mentorship support that they receive during the whole training and implementation of their projects. Greater institutional involvement and commitment from the start of the training (e.g. through signing a Memorandum of Understanding) combined with creation of implementation teams at the place of work [7] has been found to increase project ownership and support the scaling-up of new health interventions.

An important aspect of our training approach is the use of the modular nature of training that allows participants to return to their places of work after each face-to-face interactive session, thereby reinforcing theory with practice through enhancing hands-on learning. This allows the trainees to learn from familiar environments and to address real work-place problems that hamper effective delivery of services. There is also minimization of work-place disruptions since the face-to-face sessions are short (not more than 2 weeks). Similar approaches have been used to strengthen health workforce capacity in several countries including The Gambia [16], Nicaragua [17] and Liberia [18].

Our experience has shown that facilitating health workers to acquire the right competencies, for instance, in M&E and CQI, through engaging their institutions to provide them with the necessary support, including financial support and permission to be off duty to attend classes, can contribute to improvement of health service delivery within a short time. Because the project grant from MakSPH was channeled through the institutions, this increased institutional involvement and ownership of the implemented projects. The implementation of the project at the trainees’ work-place also increased prospects for sustainability because it offered additional opportunities for other staff to learn on the job as well as be motivated as project implementation progressed [18]. It is important to note, however, that despite the existence of these pointers of success, we have not yet evaluated the long-term impact of this training approach, although this is something that we intend to do in the near future.

## Challenges experienced during implementation

The implementation of the training program faced a number of challenges. For instance, it took some time before institutions could appreciate this type of training that required trainees to implement a project at the place of work. While the projects were meant to improve the skills and competences of trainees as well as enhance service delivery, some institutions viewed them as “trainees’ projects” that diverted staff from implementation of other organizational activities. In order to address this challenge, the fellowship program arranged to communicate the requirements to the institutions prior to enrolment of trainees. These preliminary discussions enabled the institutions to appreciate the program from an early start and to commit themselves to supporting the trainees in implementing their projects. This also increased the completion rates over time.

In addition, there was a problem of some of the trainees dropping out of the program before they completed the training. Our assessment shows that majority of those who did not complete the training either took jobs with other institutions mid-way through the training or were laid off by their respective institutions prior to the end of the training. This happened because our earlier approach did not involve full engagement of the institutions before trainees were enrolled. We have since instituted mechanisms to improve training retention, including conducting employment verification checks with potential trainees’ institutions prior to enrolment and admitting trainees from institutions that have shown clear understanding of the program and which commit themselves to support the trainees to undertake the training. This has improved our overall retention rate as evidenced by the increasing retention rates with each year of intake.

We realized that some of the trainees got absorbed into their routine duties and responsibilities on returning to their places of work, and this resulted in delayed project implementation particularly in those institutions that had not fully appreciated the work-based training. To address this problem, support supervision visits by program staff and/or mentors were arranged, and involved meetings with the senior management team at the trainees’ place of work, and the project teams working on the hands-on project selected by the trainees. This helped to get back the trainees on course and allowed project implementation to proceed as scheduled, and to check on the steps taken in institutionalizing the work-based problem-solving approaches. Follow-up support coupled with face-to-face facilitation after the training event has been found to be essential in assisting trainees to translate theory into practice [7,19,20] as well as increase staff confidence and morale, and informing trainers of potential weaknesses in the curriculum [19].

However, due to the fact that the program relies mainly on MakSPH faculty and external facilitators to provide on-site supervision to the trainees, sometimes planned support supervisory visits do not take place as expected. This is because majority of the faculty are also engaged in teaching and supervision of graduate students while external facilitators (who are not employees of MakSPH) tend to be too busy to afford time off their work to travel to the supported institutions as part of on-site support supervision. This has affected the number of times that facilitators could meet and interact with the trainees during project implementation. To minimize these challenges, facilitators are encouraged to make at least one supervisory visit to the institutions during the course of the training, and trainees are encouraged to keep in touch with their academic mentors through email and telephone contacts. In addition, program staffs make at least one supervisory visit to the supported institutions in the course of the training in order to assess progress in project implementation, interact with project team members and the senior management team, as well as assess the level of institutionalization of project activities. Such institutionalization plays a key role in the continuity of project activities initiated [7], and hence improve project sustainability. In future, the program intends to work with some of the program alumni to increase the pool of supervisors who can assist in supporting trainees working with institutions that are based in their respective regions.

This training is currently fully funded by CDC, which raises sustainability challenges in the event that CDC pulled out. However, this training model has got certain features that can be adopted with minimal costs to ensure sustainability: 1) No stipend is paid to the trainees since they continue to earn a salary at their place of work; and 2) The focus is on the institutions; supported institutions or individuals may be able to pay tuition for the training in future. This would cater for the training costs, which are largely facilitator fees and coordination costs while the institutions cater for the cost of the projects which are intended to address gaps in their organizations. Several institutions provide additional funds to support the Fellows’ projects. This would be similar to the FETP programs where institutions are not given funds for implementation of projects. Our future plan is to seek formal accreditation from Makerere University so that the program is offered as a post-graduate program of the University, and we anticipate that this will increase prospects for its sustainability.

Finally, as already noted, 8 out of every 10 trainees on the program were drawn from the NGO sector. This presents a missed opportunity for improving service delivery within the public health sector, considering that inadequate skills and competencies in monitoring and evaluation among public health workers continue to hamper effective health service delivery [14,15]. To address this challenge, we have made deliberate efforts to enroll targeted district local government staff, and staff working with government ministries, departments and agencies. We hope that this approach will help to increase the number of public health sector employees enrolled on the program. In addition, we are in the process of developing a formal training, mentorship and support supervision program for district-based staff to improve M&E systems at district level, given that M&E systems at district level are not functioning effectively, yet districts serve as a center of focus in Uganda’s decentralized health service delivery system.

## Limitations

The work presented in this paper has got several limitations. This paper does not include data on the longer-term impact of the training on institutional capacity, Fellows’ competencies and sustainability of the Fellows projects. The more rigorous formal evaluation of the impact and cost of the program is in plan. However, this paper provides useful experiences and process information that can inform other related programs.

## Conclusions

The modular, work-based training model strengthens the capacity of the health workforce through hands-on, real-life experiences in the work-setting and improves institutional capacity, thereby providing a practical example of health systems strengthening through health workforce capacity building. This model can be replicated in other settings in sub-Saharan Africa with similar health workforce capacity challenges.

## Competing interests

JKBM, RKW, SM work directly with the Fellowship Program that implemented the training model described in this paper.

## Authors’ contributions

JKBM: Made substantial contributions to conception and design, acquisition of data, analysis and interpretation of data, wrote the first draft of the paper, and was responsible for the final submission of the paper. RKW: Made substantial contributions to conception and design, acquisition of data, analysis and interpretation of data, contributed to the drafting of the paper, revised the draft paper for important intellectual content, and approved the version to be published. SM: Contributed to the drafting of the paper, revised it for important intellectual content and approved the version to be published. OO: Contributed to the drafting of the paper, revised it for important intellectual content and approved the version to be published. WB: Contributed to the drafting of the paper, revised it for important intellectual content and approved the version to be published. DS: Made substantial contributions to conception and design, acquisition of data, analysis and interpretation of data, contributed to the drafting of the paper, revised the draft paper for important intellectual content and approved the version to be published. All authors read and approved the final manuscript.

## Pre-publication history

The pre-publication history for this paper can be accessed here:

http://www.biomedcentral.com/1472-698X/13/8/prepub

## References

[B1] Leatherman S Ferris TG Berwick D Omaswa F Crisp N The role of quality improvement in strengthening health systems in developing countries Int J Qual Health Care 2010 22 4 237 43 10.1093/intqhc/mzq028 20543209

[B2] Frenk J The Global Health System: Strengthening National Health Systems as the Next Step for Global Progress PLoS Med 2010 7 1 e1000089 10.1371/journal.pmed.1000089 20069038 PMC2797599

[B3] Schneider D Evering-Watley M Walke H Bloland PB Training the Global Public Health Workforce Through Applied Epidemiology Training Programs: CDC’s Experience, 1951–2011 Public Health Rev 2011 33 190 203

[B4] Patel MS Phillips C Strengthening field-based training in low and middle-income countries to build to build public health capacity: Lessons from Australia’s Master of Applied Epidemiology program Aust New Zeal Health Pol 2009 6 5 10.1186/1743-8462-6-5 PMC2672090 19358710

[B5] Gaye PA Nelson D Effective scale-up: avoiding the same old traps Hum Resour Health 2009 7 2 10.1186/1478-4491-7-2 19144187 PMC2631469

[B6] Mokwena K Mokgatle-Nthabu M Mabiba S Lewis H Ntuli-Ngcobo B Training of public health workforce at the National School of Public Health: meeting Africa’s needs Bull World Health Organ 2007 85 949 954 10.2471/BLT.07.044552 18278255 PMC2636306

[B7] Bergh A-M Van Rooyen E Pattinson RC Scaling up kangaroo mother care in South Africa: ‘on-site versu ‘off-site’ educational facilitation Hum Resour Health 2008 6 13 10.11186/1478-4491-6-13 18651961 PMC2504003

[B8] Boud D Boud D, Solomon N Knowledge at work: Issues of learning Work-based learning: a new higher education? 2001 Buckingham: Open University Press

[B9] Pappaioanou M Malison M Wilkins K Otto B Goodman RA Churchill RE Strengthening capacity in developing countries for evidence-based public health: the data for decision-making project Soc Sci Med 2003 57 1925 37 10.1016/S0277-9536(03)00058-3 14499516

[B10] Kebede S Abebe Y Wolde M Bekele B Educating leaders in hospital management: a new model in sub-Saharan Africa Int J Qual Health Care 2010 22 1 39 43 10.1093/intqhc/mzp051 19951963 PMC2803009

[B11] Talbot Y Takeda S Riutort M Bhattacharyya OK Capacity-building in family health: innovative in-service training program for teams in Latin America Can Fam Physician 2009 55 613.e1 6 19509207 PMC2694088

[B12] White ME McDinnell SM Werker DH Cardenas VM Thacker SB Partnerships in International Applied Epidemiology Training and Service, 1975–2001 Am J Epidemiol 2001 154 993 9 10.1093/aje/154.11.993 11724714

[B13] Matovu JK Wanyenze RK Mawemuko S Building capacity for HIV/AIDS program leadership and management in Uganda through mentored Fellowships Glob Health Action 2011 4 5815 10.3402/gha.v4i0.5815 21364774 PMC3046003

[B14] WHO Monitoring and evaluation of health systems strengthening: An operational framework 2010 http://www.who.int/healthinfo/HSS_MandE_framework_Oct_2010.pdf. Accessed June 17, 2012

[B15] WHO Everybody’s Business: Strengthening Health Systems to Improve Health Outcomes 2007 http://www.who.int/healthsystems/strategy/everybodys_business.pdf. Accessed June 05, 2012

[B16] Conn CP Jenkins P Touray SO Strengthening health management experience of district teams in The Gambia Health Policy Plann 1996 11 64 71 10.1093/heapol/11.1.64 10155879

[B17] McEwan E Conway MJ Bull DL Malison MD Developing public health management training capacity in Nicaragua Am J Public Health 2001 91 1586 1588 10.2105/AJPH.91.10.1586 11574313 PMC1446832

[B18] Rowe LA Brillant SB Cleveland E Dahn BT Building capacity in health facility management: guiding principles for skills transfer in Liberia Hum Resour Health 2010 8 5 10.1186/1478-4491-8-5 20298565 PMC2850875

[B19] Cicciò L Makumbi M Sera D An evaluation study on the relevance and effectiveness of training activities in Northern Uganda Rural Remote Health 2010 10 1250 20170256

[B20] Ozek B Saat Z Temiz AT Kinzie B On-the-job training through follow-up visits to improve quality of family planning services Eur J Contracept Reprod Health Care 1998 3 4 201 8 10.3109/13625189809167254 10036603

